# UV-curable ladder-like diphenylsiloxane-bridged methacryl-phenyl-siloxane for high power LED encapsulation†

**DOI:** 10.1039/c8ra00063h

**Published:** 2018-02-28

**Authors:** X. X. Shang, S. Duan, M. Zhang, X. Y. Cao, K. Zheng, J. N. Zhang, Y. M. Ma, R. B. Zhang

**Affiliations:** Key Laboratory of Green Printing, Institute of Chemistry, Chinese Academy of Sciences Beijing 100190 P. R. China xinyucao@iccas.ac.cn maym@iccas.ac.cn +86-10-6253-2144 +86-10-62659019; Key Laboratory of Light Industry and Chemical Auxiliary Chemistry and Technology, Ministry of Education, Shaanxi University of Science & Technology Xi'an 710021 P. R. China

## Abstract

A UV curable ladder-like diphenylsiloxane-bridged methacryl-phenyl-siloxane (L-MPS) was synthesized from phenyltrichlorosilane, diphenylsilanediol and methacryloxypropyldimethylmethoxysilane *via* dehydrochlorination precoupling, supramolecular architecture-directed hydrolysis-condensation and end-capping reactions. The L-MPS has a condensation degree of ∼100%, and can be complete crosslinked by UV curing. XRD, TEM and molecular simulation suggest that the ladder-like molecules are close packed with a periodic distance of *ca.* 1.2 nm. The L-MPS shows transmittance of 98% and a refractive index of *ca.* 1.61 at 450 nm. The cured L-MPS with a *T*_d5%_ value of 465.5 °C showed excellent anti-yellowing and anti-sulfidation properties. The cured L-MPS film and the encapsulated LED samples were compared with those of Dow Corning OE-6630 and OE-7662. It is believed that the dense nano-ladder unit also contributes to the thermal, gas barrier and even optical properties. L-MPS shows promising potential as a high power LED encapsulant and optical coating for use in harsh environments. This work provides an approach to integrate this novel ladder structure with advanced properties.

## Abstract 2

UV-curable ladder-like polysiloxane was constructed to integrate high RI (1.61/450 nm) with high thermal stability *etc.* for high power LED encapsulation.

## Graphical abstract

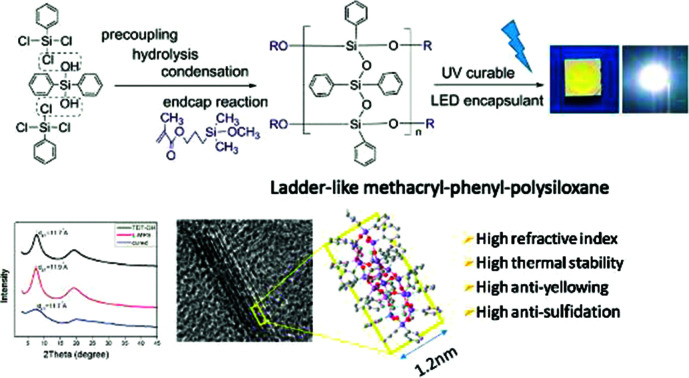

## Introduction

Polysiloxane is an important polymer species with an organic/inorganic hybrid structure that shows superior thermal stability to most polymers. With integration of high transparency, it has been widely used in electronic and semiconductor devices as optical coatings and sealants.^1–8^ With the fast development in this field, polysiloxanes with further enhanced refractive index (RI), thermal stability, anti-yellowing and gas barrier properties *etc.* are urgently needed. Furthermore, a UV-curable polysiloxane would dramatically save time and energy in the post processing programme.

One of the important applications of polysiloxanes is for light-emitting diode (LED) encapsulation. Polysiloxane based encapsulants (SiEns) have mostly replaced the traditional epoxy-resin encapsulants. The encapsulant is a key material protecting the circuits of a LED from moisture, contaminants, thermal and mechanical damage.^9–19^ The magnificent prospects of high power LEDs provide a great chance for polysiloxanes. SiEns with high transparency, high RI and durability in harsh environments are crucial for high power LEDs to gain the desired light extraction efficiency (LEE) and life time.

RI of polysiloxanes are commonly low. Increase RI of SiEn that reduces the mis-matching of RI between SiEn and the chip substrate can effectively increases LEE of LED. The RI of the intrinsic polysiloxane can be increased by introducing atoms or substituents with high molar refraction, such as halogens (except fluorine), phosphorus and aromatic groups *etc.*^20,21^ Many efforts have been made in preparation of polysiloxanes with high phenyl content.^12,17,22–30^ Taking consideration of the transparency, thermal/radiant stability and cost, polysiloxane where phenyl directly bond to Si (phenyl-siloxane) is preferred for both research and industry. Among them, the copolymer of diphenylsilanediol (DPSD) attracts a lot of attention.

Bae *et al.* reported high RI phenyl-silane by copolymerization of DPSD with R-silane (R is other than phenyl), and SiEn with high RI (∼1.58) and good thermal stability was obtain by thermal crosslinking the linear or branched oligosiloxanes bearing vinyl- and hydrido-groups through hydrosilylation.^24,25^ Although thermal curing is widely used, it usually needs hours of high temperature (∼150 °C) to accomplish the reaction. In term of time and energy saving, UV curing would be much more desired. Progress was also made in UV curable SiEn with high RI. Methacryl-siloxane with RI of ∼1.58 was reported from sol–gel condensation of DPSD and methacrylate-trimethoxysilane, but the thermal stability of this kind of polysiloxane still need to be improved.^26,27^

The RI of phenyl-siloxane would increase with the content of phenyl. However, polysiloxane or its cured film with both ultra-high phenyl content (mole ratio of phenyl/Si > 1 : 1) and good thermal stability has seldom been reported, esp. for UV-curable polysiloxane. One reason might be that it is commonly considered co-polysiloxanes with ultra-high phenyl content itself is thermally un-stable. It was reported that for methyl-phenyl-siloxane, the thermal stability increases with the phenyl content at low content but then decrease with the phenyl content.^31,32^ Actually, for different kind of polysiloxane, the relationship of phenyl content with thermal stability may still be an open question.^33^

However, there indeed long existed dilemmas for linear or branched polysiloxane to obtain both high phenyl content and high thermal stability at the same time. (1) For phenyl-siloxane resin, in order to further increase phenyl content with high RI, multi-phenyl substituted silane would be involve in the reaction. Because of the steric hindrance effect, the condensation degree of the polysiloxane (or oligosiloxane) are usually not high enough. Any remaining Si–OH or Si–OR would dramatically lower the thermal stability. (2) For the cured film, to increase the thermal stability by increasing curable group density is not applicable when high RI is desired because that means the phenyl content has to be reduced.^34^

Ladder-like polysiloxanes are best known for their higher resistance to thermal, mechanical and irradiation than the single chain counterpart because of the bridged double-chain structure.^35^ The ladder chain can be considered as densely “crosslinked” but the material is still processable. The ladder-like polysiloxanes can be dissolved or softened. The first ladder polysiloxane, ladder polyphenylsilsesquioxane, was reported by Brown in 1960s.^36^ Although there are disagreement of the regularity and configuration of the molecular structure,^37^ the ladder polysiloxane has drawn a lot of interests in both the theoretical and application research due to their unique structure and properties.^38–52^ The properties of laddered polysiloxanes can be adjusted by the side groups or the bridge-groups. The preparation method generally include equilibration polymerization,^36^ cyclo-precursor growth^40^ and supramolecular architecture-directed stepwise coupling polymerization^38^ (SCP).

We propose that integration of ultra-high phenyl content with ladder-like structure could provide a solution for the above mentioned dilemmas and lead to further enhance RI and thermal stability at the same time. In addition, the high phenyl content and dense ladder-like structure could also bring good gas barrier property for the polysiloxane.^53^

**Scheme 1:**
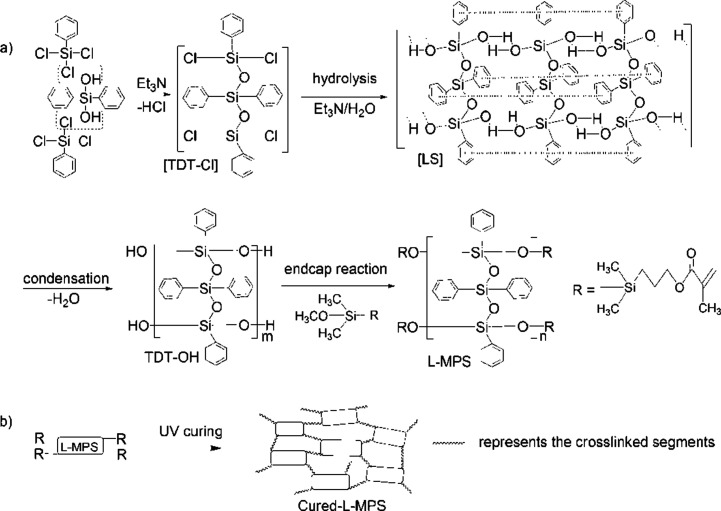
(a) The synthesis of L-MPS through a SCP method. TDT–Cl: the pre-coupled intermediate by dehydrochlorination, LS: the laddered structure by supramolecular interaction, TDT–OH: the hydrolyzed and partially condensed siloxane with silanol ends; L-MPS: the end capped ladder-like methacryl-phenyl-siloxane. (b) The cross-linking reaction of L-MPS by UV irradiation.

In this paper, we report a UV curable ladder-like diphenylsiloxane-bridged methacryl-phenyl-silxoane (L-MPS) that was prepared from phenyltrichlorosilane (PTCS) and DPSD by precoupling, step-wise hydrolysis-condensation and then followed by end-capping through methacryloxypropyl-dimethylmethoxysilane (MAOS). The synthesis processing is illustrated in Scheme 1 as SCP procedure. The L-MPS molecule has an all phenyl substituted ladder-like siloxane backbone with UV curable end groups, which would keep higher reactivity in curing process.^30^^29^Si-NMR shows that L-MPS with condensation degree (CD) of ∼100% can be obtained. The ladder-like structure was confirmed by XRD, TEM and molecular simulation with periodic distance of ∼1.2 nm. The ultra-high phenyl content L-MPS affords RI of 1.61 at 450 nm. The end-capped methacryl-group can be fully crosslinked by UV irritation reaction and the cured film shows *T*_d5%_ of 465.5 °C. The excellent anti-yellowing property and LED LEE retention upon sulphur vapour erosion of the cured film were characterized and compared with those of Dow Corning OE-6630 and OE-7662. The L-MPS shows promising potential as encapsulant for high brightness LED and high quality optical coatings, and the fast UV-curable property would also offer a time and energy saving process for large scale fabrication.

## Results and discussion

**Fig. 1:**
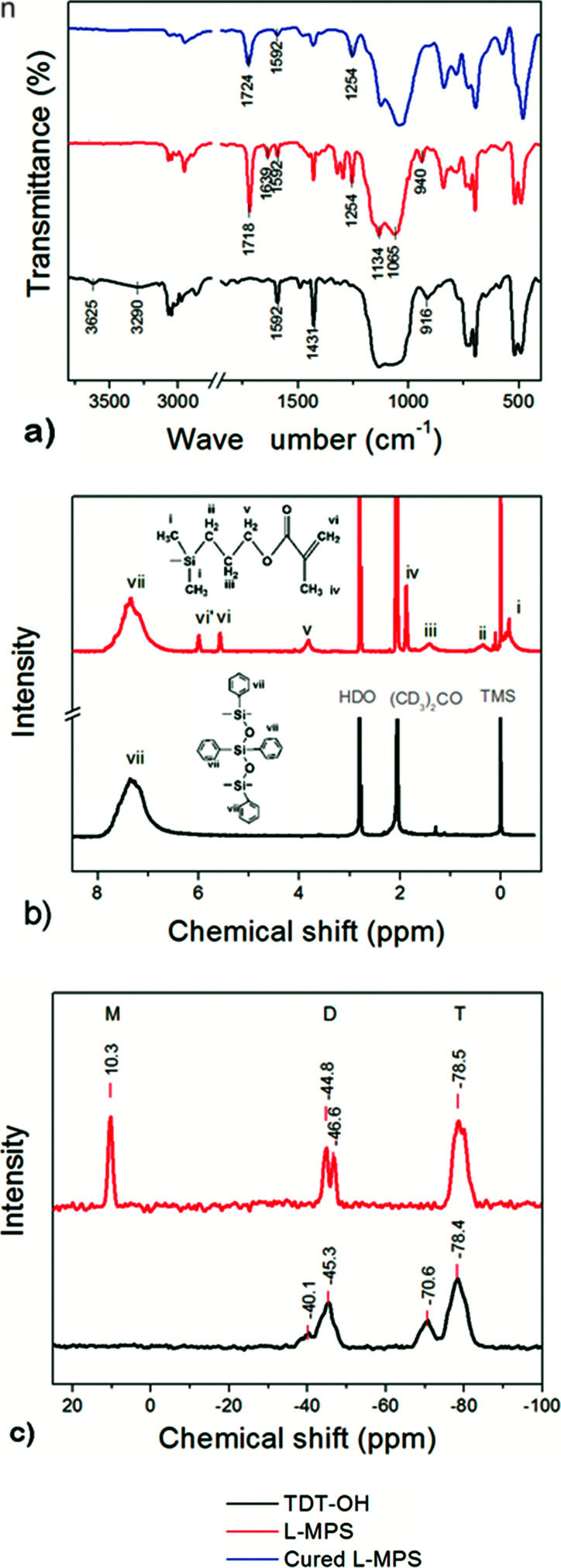
The FTIR (a), ^1^H-NMR (b) and ^29^Si-NMR (c) spectra of TDT–OH (silanol ended phenyl-siloxane, dark), L-MPS (red) and UV-cured L-MPS (blue).

The synthesis of L-MPS and the UV-curing reactions are shown in Scheme 1. The preparation of L-MPS includes dehydrochlorination-precoupling, hydrolysis–condensation and end-cap reactions. The products were monitored by FTIR, ^1^H-NMR and ^29^Si-NMR. Because of the high reactivity of Si–Cl, dehydrochlorination reaction can happen between PTCS (T) and DPSD (D) at mild condition catalyzed by Et_3_N and form the pre-coupled [TDT–Cl],^51^ which is also highly reactive in the following hydrolysis and condensation. Fig. 1 shows the FTIR (a), ^1^H-NMR (b) and ^29^Si-NMR (c) spectra of TDT–OH (silanol ended phenyl-siloxane, dark), L-MPS(red) and UV-cured L-MPS (blue).

In the FTIR spectra, the strong absorption around 1065–1134 cm^−1^ assigns to the anti-symmetric stretches Si–O bond, and the peaks at 1593 cm^−1^ and 1429 cm^−1^ are assigned to the phenyl C=C stretching and Si-phenyl stretching. After end-capping, the absorptions at 3290 cm^−1^, 3626 cm^−1^ and 916 cm^−1^ that attribute to Si–OH in TDT–OH apparently decreased. Instead, new peaks at 1719 cm^−1^ and 1256 cm^−1^ assigned to methacryl group (C=O stretching) and Si–CH_3_ (CH_3_ deformation *δ*) respectively appear in the spectrum of L-MPS.^40^

In the ^1^H-NMR spectra, the TDT–OH only shows the peak that assigned to the phenyl groups (peak vii). After the end-cap reaction, the peaks of i–vi that assigned to the MA group appear with the peak area ratio of i : ii : iii : iv : v : vi = 6.1 : 1.9 : 2.1 : 1.9 : 3.0 : 2.0 that is very close to the theoretical one (6 : 2 : 2 : 2 : 3 : 2).^26^ The peak area ratio of vii : vi = 33.5 : 2.0 which indicates that the repeat unit [TDT] number, *n*, is 6–7 referring to the formula of L-MPS in Scheme 1.

In ^29^Si-NMR spectra, the peaks at ∼−45 ppm is corresponded to the fully condensed Si (D^2^) from DPSD, and the peaks at ∼−78 ppm correspond to the fully condensed Si (T^3^) from PTCS. In the ^29^Si-NMR spectrum of TDT–OH, the weak peak at ∼−40 ppm correspond to half condensed DODP (D^1^), while the peak at ∼−71 corresponds to partially condensed OSi–OH (T^2^). After end-capping, the D^1^ and T^2^ peaks are almost disappeared and a new peak at 10.3 ppm appears corresponding to the Si from MAOS (M). The condensation degree (CD) of the polysiloxane can be calculated according to the peak area ratio of each kind of Si by eqn (1).1
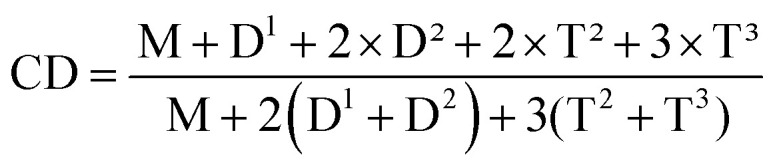


It shows that the CD of the TDT–OH is 92%, and after end-capping, CD of the L-MPS is *ca.* 100%. The peak area ratio of T : D : M in L-MPS is *ca.* 2 : 1 : 0.7 corresponding to *n* = ∼6. High CD and high reactivity of the curable function group is essential for polysiloxane to obtain high thermal stability. Any remained alkoxy- or hydroxyl groups will lower the thermal stability. The previous reported DPSD copolysiloxanes usually has the curable groups at the side of the bulky main chain that would its reactivity. The un-completely crosslinking and remaining of curable groups also lower thermal stability.

**Fig. 2:**
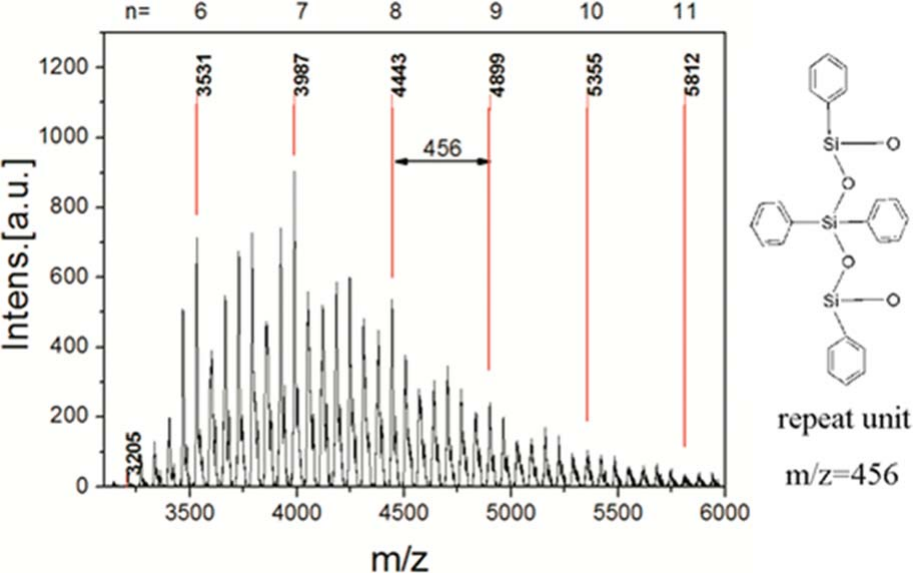
MALDI-TOF MS of L-MPS. The guideline corresponding to the molecular weight of L-MPS with TDT repeat unit of 6–11.

The MALDI-TOF MS result of L-MPS shows that the molecular weight is ∼3200–6000 corresponding to L-MPS with *n* of 5–11 (Fig. 2). It is noticed that the *m*/*z* peaks, 3531, 3987, 4443, 4899, 5355 and 5812 perfectly match the proposed molecular formula combine with Na^+^ for *n* of 6 to 11. The *m*/*z* difference between two marked peak is 456 that corresponding to one unit of [TDT]. The main peak in the spectrum is at *n* = 7, which generally agree with the results from ^1^H-NMR and ^29^Si-NMR. Of course, the other peaks between the marked peaks suggest the existence of un-regular structures (Fig. S1†).

**Fig. 3:**
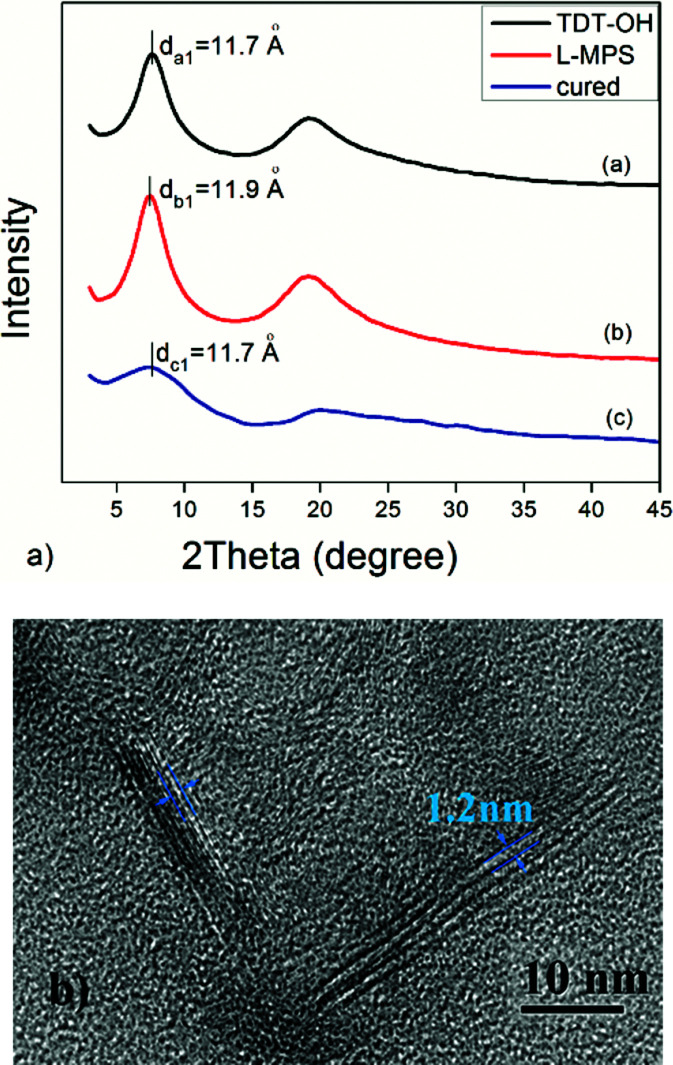
(a) XRD patterns of TDT–OH (black), L-MPS (red) and the cured L-MPS (blue). (b) HR-TEM image of L-MPS.

The ladder structure of polysiloxane is usually characterized by XRD.^35,43,44^ The typical XRD pattern of a ladder-like polysiloxane shows two halo peaks. The first peak (at lower degree) is considered corresponding to the width of the ladder molecule, and the second one corresponding to the thickness of the ladder or the amorphous phase in the material. The XRD pattern of L-MPS shows two peaks at ∼7.4° and ∼19.1° that corresponding to periodical structural distance (d) of 11.9 Å and 4.6 Å respectively (Fig. 3). The XRD curves of TDT–OH and the cured L-MPS also show the ladder feature. The XRD data are summarized in Table 1. The *d*_1_ value kept almost unchanged from TDT–OH to cured film. It suggests the ladder structure is formed during hydrolysis and condensation, and it kept in the material after curing. The molecular chain distance is mainly decided by the backbone of the ladder structure, and the flexible end group has little influence on *d*_1_. It has been verified in literatures that under mild condition, during the hydrolysis and condensation process, such pre-coupled siloxane precursors can self-assemble to a ladder-like intermediate by hydrogen bonding between Si-OHs and π–π interaction of phenyl or other aromatic groups as side group or bridge group that directing the condensation to ladder structures.^43,50,51^ Here, the forming of the ladder-like structure should also be attributed to the similar interactions between the TDT–OH as shown in Scheme 1.

**Table 1:** XRD peak data and corresponding distances

	2*θ* (°)	*d*_1_ (Å)	2*θ* (°)	*d*_2_ (Å)
TDT-OH	7.58	11.7	19.19	4.6
L-MPS	7.42	11.9	19.13	4.6
Cured L-MPS	7.57	11.7	19.91	4.5

The TEM image of L-MPS shows two kinds of parallel arranged patterns that should corresponding to the stretched molecular chains. In the left pattern, three parallel lines have the distance of 1.2 nm that agree with the *d*_1_ obtained by XRD echoes to the reported siloxane bridged triple-chain ladder polysiloxane.^38^ The pattern on the right side of the image is in larger size that two nearest parallel lines has the distance of 1.2 nm.

**Fig. 4:**
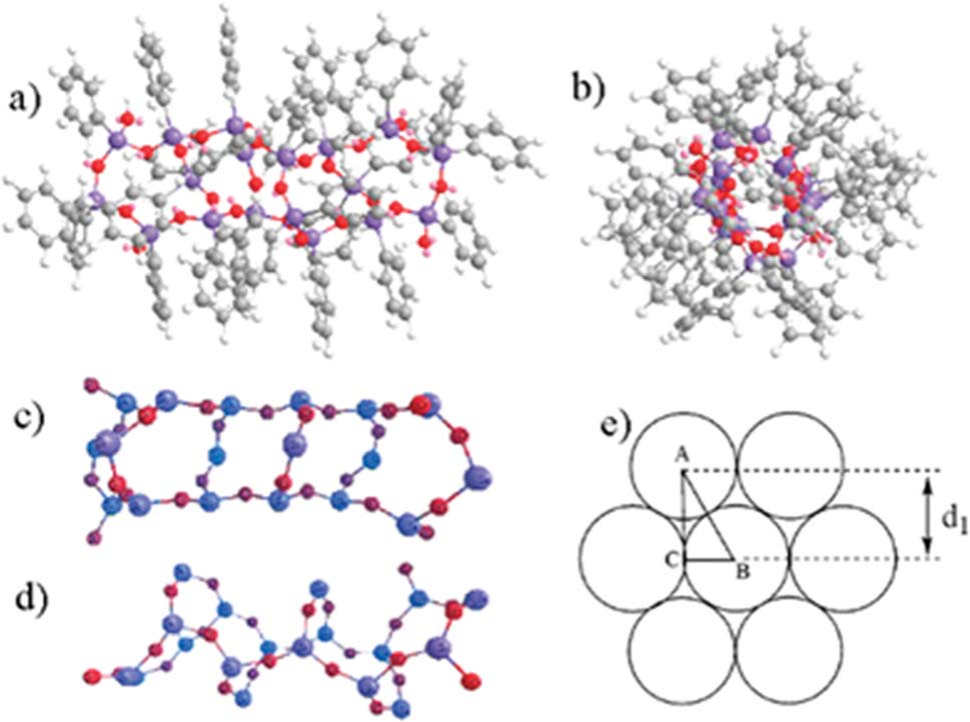
(a) The proposed L-MPS model with six repeat unit. (b) A left view of (a), along the ladder direction. (c) Top view of the ladder skeleton only showing Si (blue) and O (red) atoms. (d) Front view of the ladder skeleton. (e) Cross section view of a possible close packing of the molecules. Each cycle represent a cross section of one L-MPS molecule.

A proposed molecular model of L-MPS (*n* = 6) is shown in Fig. 4a–d. The molecular simulation shows that unlike other laddered polysiloxanes, L-MPS molecule is more like a rigid column rather than a planar molecule. The chain width from opposite phenyl to phenyl is *ca.* 13–15 Å, which general agrees with the reported silxoane-bridged triple-chain ladder polysiloxane that has a width of 13.8 Å.^43^ It is probably that the L-MPS molecules adopt a close packing stage such as shown in Fig. 4e. If the molecular width is 13.8 Å, which is actually the diameter of the molecular section. The detected layer distance *d*_1_ would be 1.2 nm, which is identical with the XRD and TEM results.

**Fig. 5:**
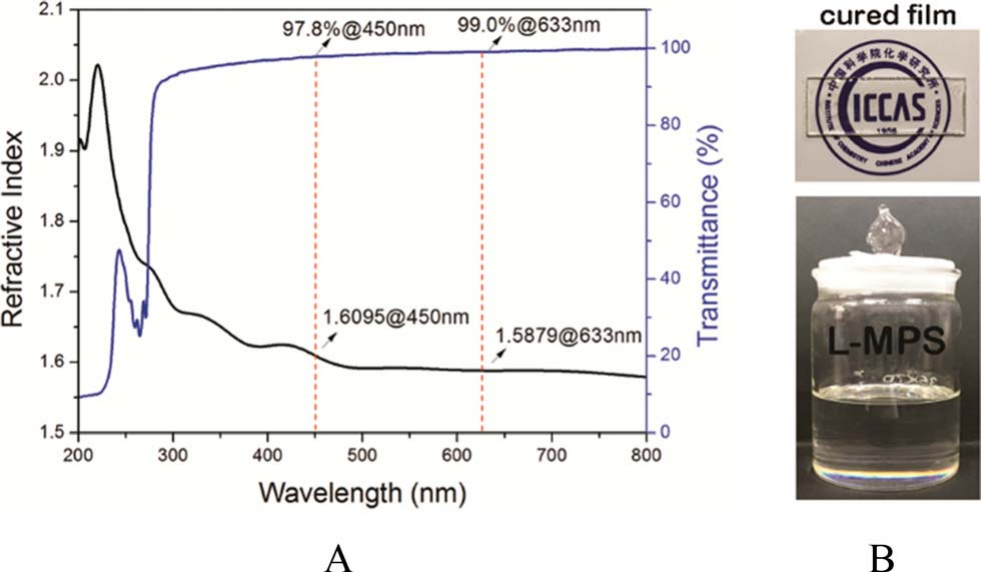
(a) The transmittance (0.1 mm thick) and refractive index (255 nm thick) curves of L-MPS. (b) The photos of L-MPS resin and the UV-cured film.

The L-MPS is a transparent colourless viscous liquid, and it can then be UV cured to form a transparent solid film. The transmittance is 98% for the 0.1 mm L-MPS film. The RI of the L-MPS is 1.61@450 nm and 1.59@633 nm (Fig. 5). The high RI attribute to the high phenyl content of the resin. The Ph/Si ratio of L-MPS is from 1.05 to 1.19 when *n* is from 5 to 11.

**Fig. 6:**
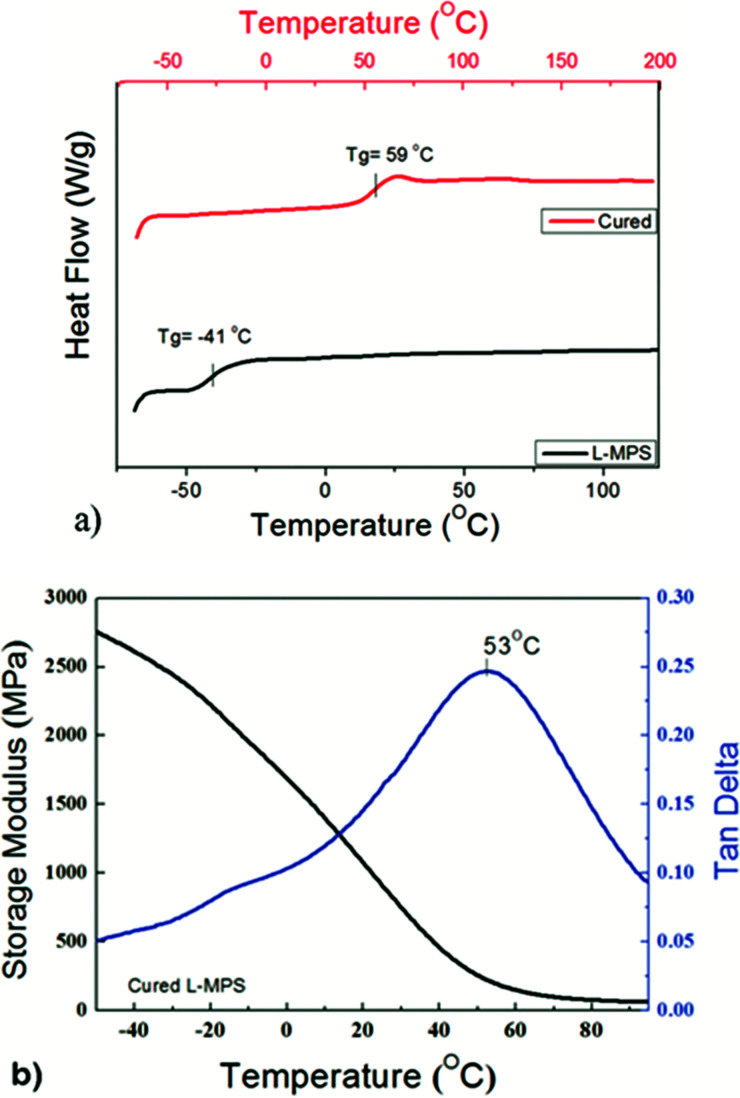
(a) DSC curves of L-MPS and the cured L-MPS. (b) The storage modulus and tan delta of the cured L-MPS measured by DMA.

The DSC scans show that L-MPS has a glass transition temperature (*T*_g_) at −42 °C. After crosslinking, the *T*_g_ of the material increased to 59 °C (Fig. 6). The storage modulus and tan delta of the cured film were measured by DMA. It shows that the *T*_g_ of the cured film is 53 °C from the tan delta curve, which agrees with the result of the DSC. The cured film has a high storage modulus both before and after glass transition. The storage modulus is 221 MPa at *T*_g_. In the rubbery state, it can maintain as 60 MPa even at 95 °C. The hardness of the film was Shore D 68 at room temperature.

**Fig. 7:**
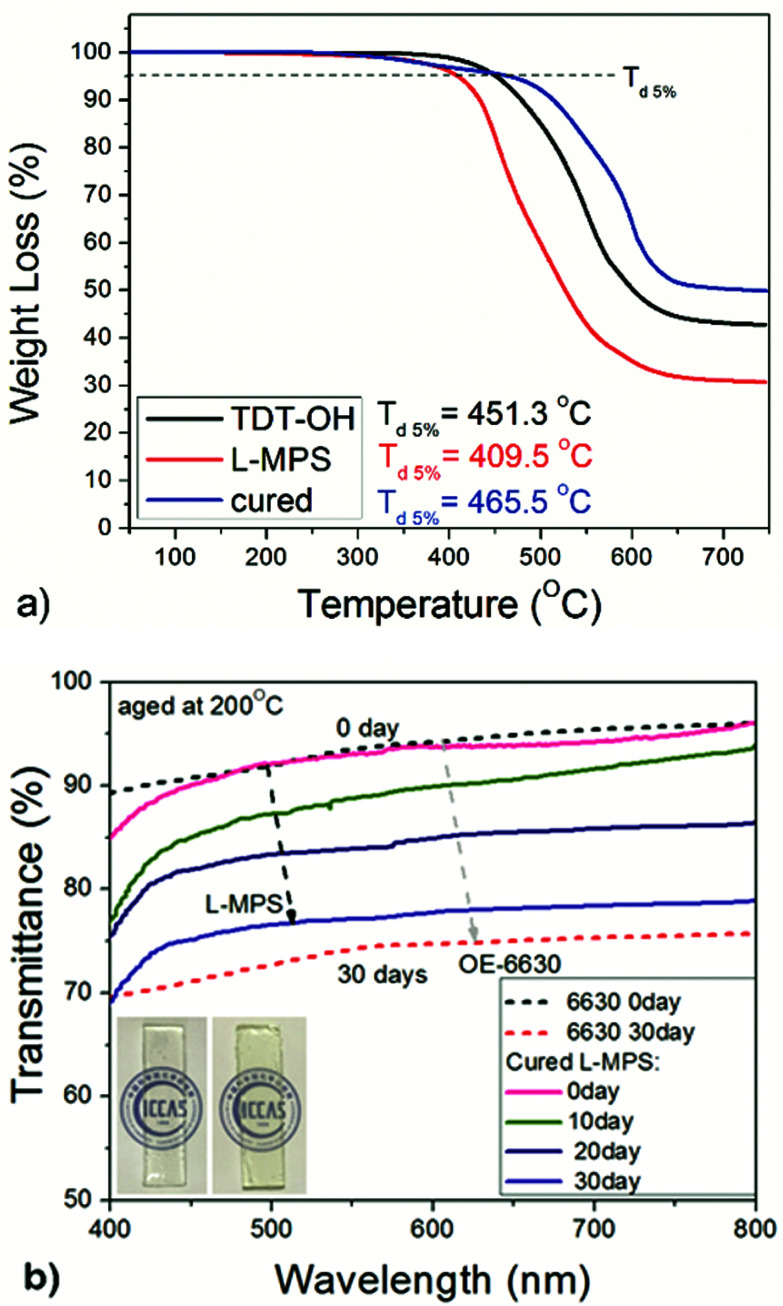
The transmittance change of cured L-MPS after aging at 200 °C for 30 days, and comparison with that of Dow Corning OE-6630. The film thicknesses are *ca.* 1.5 mm.

The TGA of the intermediate product TDT–OH was also measured and show a 5% weight loss temperature (*T*_d5%_) of 451.3 °C. XRD measurement of TDT–OH treated after 400 °C for 10 hours shows that the ladder-like backbone is quite stable and kept almost unchanged after the 400 °C treatment (Fig. S2†). After end-capping, the organic content increased would lower the *T*_d5%_, and the *T*_d5%_ of L-MPS become 409.7 °C, which is still much higher than the reported linear or branched DPSD copolymers bearing methacryl group (*T*_d5%_ no more than 371 °C).^26,27^ After curing, the film shows a high thermal stability with *T*_d5%_ of 465.5 °C. The residual at 750 °C for L-MPS and the cured film is 31% and 50% respectively (Fig. 7a). For L-MPS, the weight loss before 450 °C is mainly contributed by the decomposition of the methylpropenoyloxy-propyl (MA) group. After UV curing, the decomposition temperature of MA group dramatically increased.

The cured L-MPS film also shows good anti-yellowing property when subject to heating. Fig. 7b shows the transmittance change of the cured L-MPS film after aging at 200 °C for 720 hours. The retention was compared with that of OE-6630. Even the initial transparency of cured L-MPS was a little lower than that of OE-6630, after 720 hours aging, the transmittance was 3–4% higher than that of Dow Corning OE-6630.

To show the potential application of the L-MPS, it was coordinated into LED encapsulation. An active diluent 1,3-bis-3-(2-methylpropenoyloxy)propyl-tetramethyldisiloxane (BMAOS) was added to adjust the viscosity. The L-MPS based blend (L-MPS-B) with 30% BMAOS was applied on the 5050 LED chips, and the LEE of the as prepared sample is ∼172 lm W^−1^ which is very close with that of Dow Corning OE-7662.

**Fig. 8:**
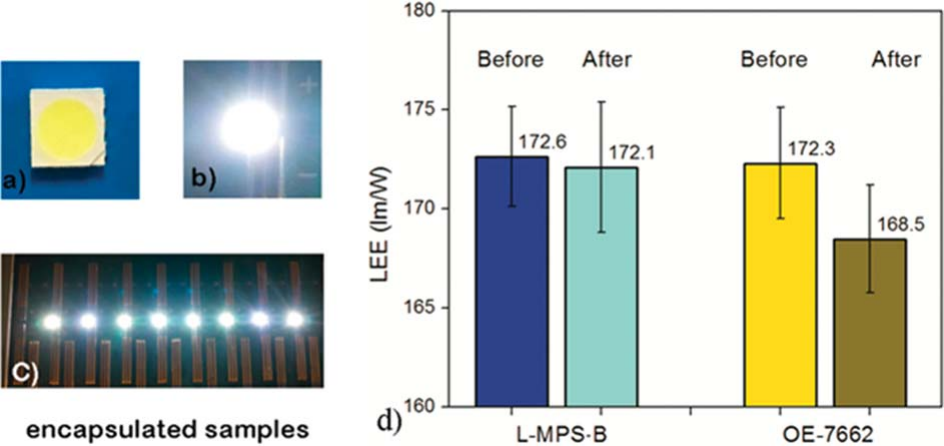
(a–c) The photos of the encapsulated sample (a) and lighted sample (b and c); (d) the LEE test before and after sulphur vapour erosion. Blue columns are for L-MPS based blend, and the yellow columns are for OE-7662.

After sulphur vapour erosion, LEE measurement shows that the L-MPS-B encapsulated sample has better sulfidation resistance. The ΔLEE (%) for L-MPS-B is −0.19%, while it is −2.10% for OE-7662 (Fig. 8). The temperature colour (*T*_c_) is also more stable for L-MPS-B encapsulated sample than that of OE-7662 (Table 2, and S2†)._._

**Table 2:** LEE results before and after sulfidation test^a^

		Before		After		ΔLEE (%)	Δ*T*_c_ (%)
		LEE (lm W^−1^)	*T*_c_ (K)	LEE (lm W^−1^)	*T*_c_ (K)		
L-MPS-B	AVG	172.64	7599	172.31	7601	−0.19	0.02
	STDEV	2.52	229	2.81	280	0.42	1.10
OE-7662	AVG	172.10	8031	168.48	8128	−2.10	1.22
	STDEV	3.27	138	2.73	151	0.50	1.33

a The typical data was from a group of six chips for each encapsulant. The complete data is listed in Table S2.

## Conclusions

This work provides an approach to integrate novel ladder polymers with advanced properties. The UV curable ladder-like diphenyl-bridged phenylsilxoane (L-MPS) was synthesized by dehydrochlorination precoupling, supramolecular architecture-directed hydrolysis/condensation and end-capping reactions from PTCS, DPSD and MAOS. The L-MPS has transmittance of 98% (0.1 mm) and RI of 1.61 at 450 nm. The CD of L-MPS is near 100% with the molecular weight of ∼3200–6000. The XRD, TEM and molecular simulation of L-MPS suggest that the ladder-like molecules are close packed with a periodic distance of *ca.* 1.2 nm. The cured L-MPS hold up high storage modulus at rubber state (60 MPa at 95 °C) measured by DMA. The *T*_d5%_ of the cured film is at 465.5 °C, and the film shows excellent anti-yellowing ability with 3–4% higher transmittance retention compared to that of Dow Corning OE-6630 after aged at 200 °C for 720 hours. The LEE measurement of the LED chips encapsulated by L-MPS blend also showed better sulphur vapour resistance and less LEE and *T*_c_ decline than those of OE-7662. The dense nano-ladder unit also contribute to the above advanced properties. L-MPS and the cured material with the combination of high RI, high thermal stability, anti-yellow and anti-sulfidation property shows promising potential as high power LED encapsulant and optical coating for harsh environment.

## Experiment

### Materials

Diphenylsilanediol (DPSD 98%), phenyltrichlorosilane (PTCS, 98%), 2-hydroxy-2-methyl-1-phenyl-1-propanon (photoinitiator-1173, 98%), Irganox-1076 (>98%) and Irgafos-168 (>98%) were purchased from TCI; methacryloxypropyldimethylmethoxysilane (MAOS, 98%), 1,3-bis-3-(2-methylpropenoyloxy)propyl-tetramethyldisiloxane (BMAOS, 95%) was purchased from Gelest; the phosphor YGG-530 was from China Minmetals Corporation; tetrahydrofuran (THF), methylbenzene, magnesium sulfate, sodium chloride, hexane, triethylamine (Et_3_N), sulphuric acid were all AR grade and purchased from Beijing Chemical Works.

### Preparation

#### 1 Synthesis of L-MPS

(a) Precoupling: DPSD was added into PTCS by droplet and reacted at room temperature under the catalysis of Et_3_N in THF. The mole ratio of PTCS : DPSD : Et_3_N = 2 : 1 : 2. The product is labeled as [TDT–Cl]. (b) Hydrolysis and condensation: to the above solution, water and Et_3_N was added by droplet based on the mole ratio of water : Et_3_N : [TDT–Cl] = 5 : 4 : 1. The reaction was carried out at 0 °C for 2 hours. After filtration, the temperature was increased gradually to 80 °C. During this process, toluene was added and the remaining water was removed by azeotropic distillation. TDT–OH was obtained. (c) End capping: MAOS was added to the above TDT–OH solution based on weight ratio of 3 : 5. After trace of sulphuric acid was added, the reaction was carried out at 40–60 °C for 6 hours. Then excessive MAOS and solvents were removed by vacuum evaporation.

#### 2 UV curing of L-MPS

The cured film was obtained by mold casting the L-MPS with of initiator-1173 and irritated in an inert gas charged box equipped with UV lights (365 nm, 800 W) for 15 minutes. For the sample of thermal aging, L-MPS with 0.4 wt% of Irganox-1076, 0.1 wt% of Irgafos-168 and 1 wt% of initiator-1173 was used. Dow Corning OE-6630 was casted and thermal cured at 80 °C for 1 hour and 150 °C for 2 hours as a comparison sample.

#### 3 LED encapsulation

The L-MPS and BMAOS (2 : 1) was mixed with 15 wt% phosphor, 0.4 wt% of Irganox-1076, 0.1 wt% of Irgafos-168 and 1 wt% of initiator-1173 and de-foamed at the room temperature. The mixture was then applied on the dry lensless-5050 lead-frame with flat surface and UV-cured. Dow Corning OE-7662 with 15 wt% phosphor was applied for comparison which was crosslinked at 80 °C for 1 hour and 150 °C for 2 hours as a comparison sample.

### Characterization

Fourier transform infrared spectrometry (FTIR) spectra were recorded by a BrukerEQUINOX55 spectrophotometer. ^29^Si-NMR spectra were performed on Bruker 300 MHz instruments (DMX300). The molecular mass was measured by MALDI-TOF MS spectrometer (Bruker Autoflex III). X-ray diffraction (XRD) analysis was recorded on Empyrean. The transmission electron microscope (TEM) image was captured by TEM, JEM-2200FS, JEOL, Japan. The thermal gravimetric analysis (TGA) was performed on a Perkin-Elmer/Pyris 1 TGA by the heating rate of 20 °C min^−1^ in nitrogen atmosphere. *T*_g_ of the products were performed by differential scanning calorimetry (DSC, Q2000, TA Instruments) at a heating rate of 20 °C min^−1^. Dynamic mechanical analysis (DMA) was performed on DMA800-TA at a heating rate of 3 °C min^−1^. The optical transmittance was measured by an ultraviolet-visible spectrophotometer (UV2600, SHIMADZU). The refractive index (RI) of the L-MPS resin was measured by an Ellipsometry (J. A. Woollam Co., Inc., RC2) and Kramers–Kronig compliant. The shore hardness was obtained by shore durometer (LX-A/D). The LEE was detected by spectrophotocolorimeter with the LED spec analysis system (STC 4000, Everfine photo-E-Info Co., LTD.). The gas barrier property was demonstrated by the LEE and *T*_c_ change in the anti-sulfidation test, in which samples and 1.34 g sulphur powder were kept at 85 °C for 4 hours in a 800 ml sealed container. The chemical formula were drawn by ChemDraw Ultra 10.0 and the molecular simulation was carried out by Chem3D Ultra 10.0.

## Conflicts of interest

There are no conflicts of interest to declare.

## Supplementary Material

RA-008-C8RA00063H-s001
