# Supplementary material for: UV-curable ladder-like diphenylsiloxane-bridged methacryl-phenyl-siloxane for high power LED encapsulation
Source: RSC Adv. 2018 Feb 28;8(17):9049–56. doi: 10.1039/c8ra00063h (PMC9078592; doi:10.1039/c8ra00063h)
Supplement: RA-008-C8RA00063H-s001 [file RA-008-C8RA00063H-s001.pdf]

Electronic Supplementary Material (ESI) for RSC Advances.
This journal is © The Royal Society of Chemistry 2018

Supporting information

UV-curable ladder-like diphenylsiloxane-bridged methacryl-phenyl-siloxane for high power LED encapsulation

Xinxin Shang [a, †], Shan Duan [a, b, †], Min Zhang [b], Xinyu Cao [a,*], Kun Zheng [a], Jingnan Zhang [a], Yongmei Ma [a, *] and Rongben Zhang [a]

[a] Key Laboratory of Green Printing, Institute of Chemistry, Chinese Academy of Sciences, Beijing 100190, P. R. China; [b] Key Laboratory of Auxiliary Chemistry & Technology for Chemical Industry, Ministry of Education, Shaanxi University of Science & Technology, Xi'an 710021, P. R. China.

Table S1. The theoretical data m/z for L-MPS with different repeat unit (n) and those found in MALDI-TOF MS perfectly match theoretical ones

	Theoretical data† (m/z)		Found in MALDI-TOF MS (m/z)
n	L-MPS	L-MPS complex with Na^+	the marked peaks in the graph.
1	1228.5	1250.5	--
2	1684.6	1706.6	--
3	2140.7	2162.7	--
4	2596.8	2618.8	--
5	3052.9	3074.9	--
6	3509.0	3531.0	3531
7	3965.1	3987.1	3987
8	4421.2	4443.2	4443
9	4877.3	4899.3	4899
10	5333.4	5355.4	5355
11	5789.5	5811.5	5812

† The theoretical data were obtained from ChemDraw Ultra 10.0 by put in the formula of the molecules.

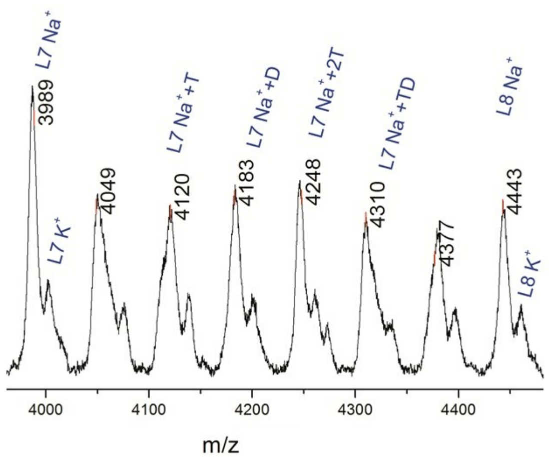

Figure S1. The enlarged MALDI-TOF MS spectrum between L-MPS of n=7 (L7) and n=8 (L8).

The enlarged MALDI-TOF MS spectrum between L-MPS of n=7 (L7) and n=8 (L8) shows that the peaks between L7 and L8 are mainly correspond to the proposed ladder structure with an additional T, D, TT or TD group. At this stage, it is hard to define how the additional groups attach to the main chain, but the combination of the characterization results, including those from XRD and TEM, support the ladder-like structure of L-MPS.

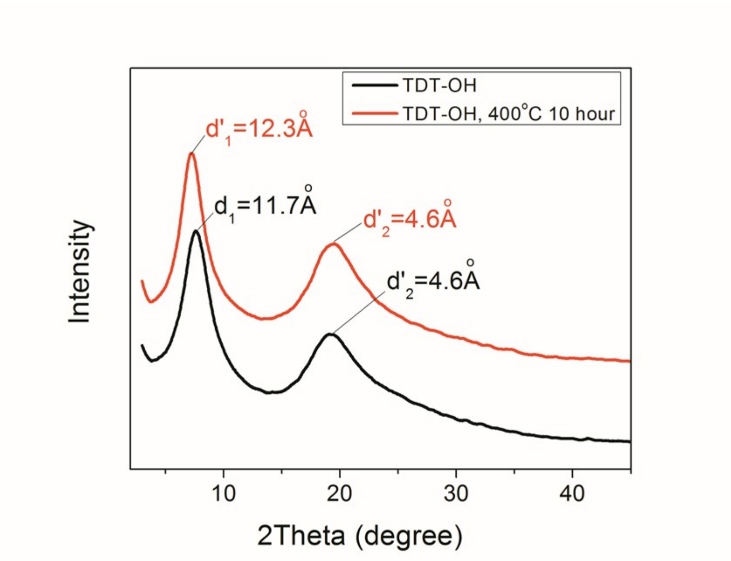

Figure S2. The XRD curves of the TDT-OH before and after treated at 400°C for 10 hours.

The XRD measurement shows that after 400°C for 10 hours, the peaks represent the ladder structure kept almost unchanged.

Table S2. The comparison of anti- Sulfidation of L-MPS-B with OE-7662.

sample	Item	before		after			
		LEE (lm/W)	Tc(K)	LEE (lm/W)	Tc(K)	∆LEE(%)	∆Tc (%)
Ladder	1	174.39	7332	174.93	7238	0.31	-1.28
	2	174.99	7334	175.29	7377	0.17	0.59
	3	169.27	7578	169.36	7564	0.05	-0.18
	4	174.47	7771	173.64	7748	-0.48	-0.30
	5	169.75	7700	168.7	7652	-0.62	-0.62
	6	172.95	7881	171.91	8030	-0.60	1.89
	**Average**	**172.64**	**7599**	**172.31**	**7601**	**-0.19**	**0.02**
	STDEV	2.52	229	2.81	280	0.42	1.10
OE-7662	1	172.93	8093	169.22	8280	-2.15	2.31
	2	175.7	7910	170.47	8082	-2.98	2.17
	3	169.5	7985	166.06	8098	-2.03	1.42
	4	176.11	7852	172.6	7892	-1.99	0.51
	5	169.34	8140	165.93	8307	-2.01	2.05
	6	169.04	8206	166.62	8113	-1.43	-1.13
	**Average**	**172.10**	**8031**	**168.48**	**8128**	**-2.10**	**1.22**
	STDEV	3.27	138	2.73	151	0.50	1.33
